# Obstetric referral practices and health system factors in public health centres of Addis Ababa, Ethiopia: A mixed-methods study

**DOI:** 10.1371/journal.pone.0342872

**Published:** 2026-08-25

**Authors:** Amaha Haile Abebe, Rose Mmusi-Phetoe

**Affiliations:** 1 Yeroam Consultancy, Addis Ababa, Ethiopia; 2 University of South Africa, Pretoria, South Africa; LMU München: Ludwig-Maximilians-Universitat Munchen, NEPAL

## Abstract

**Objective:**

To determine the rate of obstetric referral and explore contextual factors influencing referral practices in public health centres of Addis Ababa, Ethiopia.

**Design:**

Explanatory sequential mixed-methods study.

**Setting and period:**

Fifty public health centres in the Addis Ababa City Administration, January–April 2021.

**Methods:**

Delivery and referral registers from 50 health centres were reviewed retrospectively for 12 months (8 July 2019–7 June 2020). Facility observations and interviews with maternity unit heads were conducted in all selected centres. In-depth interviews were conducted with 20 midwives and 13 health centre managers. Quantitative data were analysed descriptively, and qualitative data were analysed thematically using Colaizzi’s method.

**Results:**

Only 13 (26%) of the 50 health centres have met all eight standards assessed for functionality of the referral system. The main gaps of the referral system were a lack of ambulance (12%), absence of formal agreement with referral centers, and lack of timely case-by-case feedback from referral centers(64%). The overall obstetric referral rate was 32%, with substantially higher referral rates in health centers without caesarean section (CS) services compared with those providing CS (39% vs 21%). Qualitative findings indicated that high referral rates were associated with limitations in the predictive capacity of the partograph, variability in providers’ clinical skills, and risk-averse practices driven by accountability concerns related to maternal and perinatal outcomes.

**Conclusion:**

Improving functionality of referrals and reducing unnecessarily high referrals requires adopting and training providers on the new WHO labour guide and partograph, improving referral feedback, and clarifying accountability mechanisms.

## Introduction

Most pregnancies and births are uneventful. However, around 15% of all pregnant women will develop a potentially life-threatening complication that calls for skilled care, and some will require a major obstetrical intervention to survive [1]. Consequently, about 15% of all pregnant women are expected to develop life-threatening obstetric complications, and must be promptly referred from lower levels of care to facilities capable of providing comprehensive emergency obstetric care, including caesarean section delivery [2,3].

Effective referral systems are essential for reducing delays in accessing appropriate care. However, referral capacity at the primary care level remains weak in many sub-Saharan African countries. Analysis of service availability data from five countries showed that only 38% of primary care facilities had adequate referral capacity [4].

The World Health Organization (WHO) quality-of-care standard identifies a functional referral system as one of the eight core domains of quality maternal and newborn care [5].The standards emphasize that every woman and newborn with conditions beyond the capacity of a facility should be appropriately and promptly referred. The standard includes timely assessment at admission, during labour, and in the early postnatal period; rapid decision-making and a functioning referral system. A functional referral system that has standard referral forms and procedures, a list of pre-established referral networks, a readily available ambulance, and effective communication and feedback between referring and receiving facilities [5].

The WHO standard underscores that providers’ skills in timely and accurate identification of obstetric complications and rapid referral decision-making are critical to an effective referral system [5]. However, studies reported that a significant proportion of providers at primary health care facilities lack such skills [6].

A referral decision must be timely and appropriate. Unnecessary referrals burden higher-level facilities and impose avoidable costs on families. The WHO estimates that 10–15% of pregnant women develop complications requiring caesarean section [3]. Primary health care facilities without Cesarean Section capability would therefore be expected to refer approximately 15% of pregnant women and women in labour. However, reported referral rates often exceed this range, 28–63% [7–9].

In Ethiopia, 73% of women with obstetric complications admitted to health centres were referred, suggesting over-referrals and underutilization of available services at health centres [10]. Another study in Ethiopia also reported that only 10% of all the obstetric referrals at referral centers were appropriate referrals [11].

WHO standard identified reliable transport—typically ambulances available 24 hours a day—is a critical component of effective referral systems [5]. In Ethiopia, only 17% of health facilities have a dedicated, functioning ambulance [10]. Similar challenges have been reported in other developing countries [12]. Beyond availability, ambulance management—including fuel supply, driver availability, and prioritization for emergencies—directly affects referral timeliness [13].

As per the WHO standard, effective referral systems also depend on effective communication and feedback mechanisms. Although most Ethiopian facilities assign staff to accompany referred women, advance notification and systematic feedback from referral hospitals remain uncommon [10]. Comparable gaps have been documented in South Africa [14].

Many studies assessed the common obstetric indications and appropriateness of referrals from clinical grounds at primary health care facilities. However, there is limited data on the functionality of the referral system at public health facilities using WHO quality standards, and there is scanty qualitative data on contextual factors that drive referrals at primary health care facilities. This study assessed the rate, functionality, and contextual drivers of obstetric referral in public health centres in Addis Ababa City.

## Materials and methods

### Study setting

Ethiopia has a three-tier health care delivery system organized into primary, secondary, and tertiary levels. The primary level includes health posts, health centers, and primary hospitals that provide preventive, promotive, and basic curative services to the community. The secondary level consists of general hospitals that offer more advanced diagnostic and treatment services and receive referrals from primary facilities. The tertiary level includes specialized hospitals that provide highly advanced medical care, teaching, and research services. These three levels are connected through a referral system to ensure continuity and quality of health care.

There are ten sub-cities in the Addis Ababa city Administration with health offices responsible for managing the health centers. The city had a total of 97 health centers providing maternal health care services during the study period. The basic health centers in Ethiopia provide basic obstetric and newborn care services, including vacuum-assisted delivery. Comprehensive health centers, in addition, provide comprehensive obstetric care, including cesarean section. The health centers in Addis Ababa refer out women with life-threatening obstetric conditions to hospitals in their catchments. The focus of the study was referrals from the health centers to the hospitals (referral-out).

### Study design

An explanatory sequential mixed-methods design was used. First quantitative data was collected using retrospective record review and structured observation, and interviews with midwives to assess functionality of the referral system along eight WHO standards. Once the quantitative data were analyzed, a qualitative phase was conducted using in-depth interviews to explain the quantitative findings. The quantitative and qualitative data collection was started on January 1, 2021, and completed on April 28, 2021.

### Quantitative phase

#### Quantitative data sources and sampling.

The study was conducted in fifty public health centres that were randomly selected from the 97 health centres providing maternal health services in Addis Ababa during the study period. The study collected data in only fifty health centres due to time and budget constraints.

Five health centres were selected from each of the city’s ten sub-cities using a lottery method. Delivery, referral, and operating theatre registers were reviewed during the above-mentioned study period. Record review covered registers for the Ethiopian Fiscal Year 2012 (8 July 2019–7 June 2020). Facility interviews and observations were conducted using a structured checklist to assess the functionality of the referral system.

#### Quantitative data collection.

Four trained midwives who have a master’s degree in reproductive health or public health extracted data using a structured checklist from the labour and delivery and referral registers, capturing data on service volume, mode of delivery, operative vaginal delivery, referrals, and caesarean section procedures. The register review included counting data on the total number of women presented for labour and delivery, the total number of women who had childbirth in the same health facility, and the total number of women referred to a higher-level facility.

The trained midwives who have master’s degree and research experience conducted interviews with head midwives at the maternal health services, and observations at the 50 health centers using a structured questionnaire to assess if the health facility meets eight standards defined for a functional referral system. The principal investigator supervised data collection and conducted daily data quality checks.

The study did not review individual obstetric and newborn care delivery records of women referred to collect data on the appropriateness of referrals.

#### Quantitative data analysis.

Quantitative data were entered into EpiData, then transferred to SPSS version 20 for analysis. Descriptive statistics (means, medians, proportions, ranges) were used to analyze quantitative data.

Obstetric referral in this study refers to referrals out, i.e., obstetric referrals from the study health centers to higher-level health facilities that provide comprehensive obstetric and newborn care services.

Obstetric referral rates were calculated for facilities for the Ethiopian Fiscal Year 2012 (8 July 2019–7 June 2020). Referral rate was calculated by dividing the total number of women referred for obstetric and newborn care-related cases to higher level facilities from the health centres by the total number of obstetric admissions (delivered in the same health facility and referred to higher level care) by the health centres multiplied by 100 [15].

Availability of a functional referral system was assessed using eight standards adapted from the WHO maternal and newborn care standards [5]. The eight referral care standards assessed include availability of a functional ambulance, a standard referral form, a referral register, a list of network referral centers, a formal agreement with referral centers, availability of functional communication means (telephone and radio) for communication on referrals, existence of feedback on referrals, and accompanied referral to referral centers.

A composite index called functional referral was constructed from the eight standards using a summative score. Each of the eight standards had a yes and no response option, and the standard was scored ‘1’ when the health center met the standard and ‘0’ when the health center did not meet the standard. A summative score was calculated for each health center on a scale of 8 points, and a health center was considered to have a functional referral system when it met all 8 standards.

### Qualitative phase

#### Qualitative data source and sampling.

A total of 33 people, including 20 midwives and 13 health centre managers, who are directly involved in maternity care and service management, participated in the in-depth interview. The in-depth interview participants were selected using purposive sampling, where people who have in-depth information were purposely selected for the interview.

#### Qualitative data collection.

In-depth interviews were conducted by the principal investigator using a semi-structured guide. The in-depth interviews were audio-recorded with consent and supplemented by field notes. In-depth interviews were conducted in a venue that ensures the privacy and confidentiality of the study participants.

#### Qualitative data analysis.

In-depth interviews were tape-recorded. The principal investigator, who is a native speaker of the language of the interview, transcribed and translated the in-depth interview tape records into English. The qualitative data were analyzed using Colaizzi’s seven-step process for phenomenological data analysis [16]. The qualitative data coding was assisted by Atlas ti 23, software for computer-aided qualitative data coding. The two discussion themes were 1) availability of a functional referral system and 2) Reasons for the high obstetric referral rate. Three sub-themes emerged under the first theme, which include lack of referral guidline, mismanagement of ambulances, and lack of feedback on referrals. Similarly, three sub-themes emerged under the second theme, which includes the low specificity of the partograph, lack of clinical skill, and risk-averse practices driven by accountability pressures.

### Ethical considerations

The research protocol was reviewed and approved by the Research Ethics Committee of the Department of Health Studies of the University of South Africa. The research protocol was again reviewed and approved by the Ethical Review Committee of the Addis Ababa city administration health office. Once the research protocol had been approved by the ethical review committees, support letters were written from the Addis Ababa city administration health office and sub-city health offices to study health facilities. The funders had no role in study design, data collection and analysis, decision to publish, or preparation of the manuscript.

Written informed consent was obtained from all study participants, and interviews were conducted in a setting that ensured privacy and confidentiality. The study did not collect or access data that identifies individual clients’ identities.

### Limitations of the study

A major limitation of the study was that it did not include a review of individual clients’ clinical charts to document obstetric profiles and appropriateness of referrals based on clinical guidelines.

## Results

### Profile of health centers and study participants

All the 50 health centres were providing antenatal, labour and delivery, postnatal, family planning, and abortion care services. The labour and delivery service was provided 24 hours a day and seven days a week in all 50 health centres. Only 12 (24%) of the health centres were providing caesarean section delivery during the study period.

Half (25) of the health centres were serving only urban residents, while the other half (25) were serving both urban and rural residents. As per the Ethiopian MoH standards, an urban health centre is expected to serve a maximum catchment population of 40 000 people, and 32 (64%) of the health centres were serving a catchment population of 40 000 or less. The rest, 18 (36%) health centers, were serving a catchment population of more than 40 000 people, which was above the MoH standard. The mean number of catchment population per health centre was 38 703 people (the standard deviation was 17 999), and the median was 35 669 people.

An in-depth interview was conducted with a total of 33 participants, 20 of whom were midwives who were maternity unit heads, and 13 were managers. Of the managers, 10 were medical directors, and three were quality department heads. Of the 33 participants, there were 27 females and 6 males. Ten participants were under the age of 30 years, while 13 were 35 years and older. Half of the participants had 5−9 years’ work experience Table 1.

**Table 1:** Demographic characteristics of in-depth interview participants (N = 33).

Demographic characteristics	Midwives Number	Managers Number	Total Number	Percent
Gender				
Female	16	11	27	81.8
Male	4	2	6	18.2
Age				
< 30 Years	9	1	10	30.3
30–34 years	8	2	10	30.3
≥ 35 Years	3	10	13	39.4
Work Experience				
< 5 Years	4	0	4	12.1
5–9 years	14	3	17	51.5
≥ 10 Years	2	10	12	36.4

### Functionality of referral systems

Overall, only 13 of the 50 health centres (26%) met all eight standards for a functional referral system assessed. Most health centres had a functioning ambulance (84%), reliable communication mechanisms (92%), standard referal forms and an up-to-date list of referal system (96%). However, 12% of health centers don ot have their own ambulance, 34% of health centres do not have a formal agreement with referal centres, and 64% of health centres do not provide timely, case-by-case feedback from referral hospitals Table 2.

**Table 2:** Percentage distribution of health centres with functional referal system (N = 50).

Functioning Referral System	No (%)	Yes (%)
Owned a functioning ambulance for the emergency transport of women and newborns to referral facilities	16	84
Had a functioning communication method (a mobile phone, land line or radio) for referrals and consultation on complicated cases	8	92
Had an up-to-date list of network facilities that provide referral obstetric and newborn care	4	96
Had formal agreements, communication arrangements, and a feedback system with referral centre(s)	34	66
Had a standardized referral form that enabled them to document relevant clinical information and the reason for referral	4	96
Had appropriate documentation or register for referred cases	10	90
Always assigned professionals to accompany cases to referral facilities	10	90
Regularly received case-by-case, timely feedback	64	36
Had an effective referral system (met all eight standards for a functional referral system)	74	26

### Obstetric referral rates

The overall obstetric referral rate was 32%. Referral rates were higher in facilities without caesarean section (CS) services (39%) than in those providing CS (21%). Only five facilities reported referral rates below the expected 15% threshold Table 3.

**Table 3:** The mean number of women provided labour and delivery care and obstetric referrals during Ethiopian Fiscal Year (EFY) 2012 (8 July 2019–7 June 2020) (N = 50).

Volume of service	Mean	Median	Standard Deviation	Minimum	Maximum	Sum (total)
Catchment population	38,703	35,668	17,999	12,440	98,000	193,5136
Expected number of pregnancies in the catchment population	890	820	413	286	2,254	44508
Total number of labour and delivery cases seen (delivered plus referred)	856	807	616	159	3,338	44,283
Total number of deliveries attended	605	521	457	72	2071	30,270
Total number of obstetric and newborn cases referred to higher-level facilities	280	241	223	58	1491	14,013

### Qualitative findings

#### Theme 1. Availability of functional referral system.

Three sub-themes emerged under this theme, which include lack of referral guidline, mismanagement of ambulances, and lack of feedback on referrals

Lack of referral guidline: Midwives reported lack of guidline for obstetric referrals. Midwives believe a referral guideline that lists obstetric cases and conditions that should be referred from the health centre to the hospital could standardize referral and reduce unnecessary referrals. A manager said:

*“Referral facilities sometimes refuse to accept referred cases, stating the case does not fulfil the criteria for referral. We do not have clear guidelines on referral that could standardize referral, and reduce unnecessary referrals”.*

Mismanagement of the ambulance: Midwives reported that most health centres have ambulances, and the problem is with the management of the ambulances. Midwives noted that the use of ambulances for administrative purposes, a lack of budget for fuel, or drivers’ absence from the workplace sometimes cause delays in referrals. A midwife said:

*“We have ambulance. The problem is fuel. The management also uses the ambulance for admin purposes, which creates a delay in referrals.”*

Lack of feedback on referrals: Midwives and managers unanimously said there was no timely feedback on cases referred to hospitals. Most midwives believed immediate case-by-case feedback could help to improve the quality of care. A medical director said:

*“We do not have case-by-case immediate feedback that could have improved accountability, and the quality of care…”*

#### Theme 2: Reasons for the high rate of referrals.

Three sub-themes emerged under this theme, including the low specificity of the partograph, lack of clinical skill, and risk-averse practices driven by accountability pressures.

Low specificity of the partograph: Midwives attributed high referrals to the limited ability of the partograph to accurately identify women with life-threatening obstetric complications. Midwives noted that most women, especially the primigravida, do not progress at a rate of one centimeter per hour as assumed in the partograph in the active first stage of labour and cross the action line. Many of these women who crossed the action line are diagnosed with prolonged labour and referred to the hospital to subsequently deliver normally at referral hospitals. A midwife said

*“Most women cross the action line, yet they deliver normally at the hospital. The partograph needs to be improved for its ability to detect real problems.”*

Providers lack of clinical skills: Midwives identified providers lack of skill, particularly in cervical dilatation assessment, to contribute to misdiagnosis of the latent stage of labour as an active stage of labour, and this contributes to unnecessary and high referrals. A midwife said:

*“The problem is a lack of skill in the estimation of cervical dilation. Women in the latent phase of labour are diagnosed as active first stage of labour and started on a partograph. Then the women will cross the action line and be referred for prolonged labour”.*

Risk-averse practices driven by accountability pressures: Managers emphasized risk aversion driven by strict accountability for maternal and perinatal deaths, which encouraged early referrals.: A manager said

*“Providers prefer to refer early because of strict accountability for maternal and perinatal deaths.”*

## Discussion

A functional referral system is key to preventing maternal and perinatal mortality. Obstetric complications are unpredictable and progress rapidly to become severe and life-threatening. Therefore, women with complications at lower levels of care should be referred to and reach referral centres that can provide emergency interventions [2]. The WHO quality standard identified the availability of a functional referral system as one of the eight domains of quality of obstetric and newborn care [5]. However, only a quarter of the health centres meet all eight standards of the functional referral system assessed. About 12% of the health centers do not have their own ambulance, 34% do not have formal agreements with referral centres for referrals, and 64% of the health centres do not receive timely feedback from referral facilities.

Availability of adequately equipped transport services that operate 24/7 is a critical component of a functional referral system [5]. In this study, though the majority (84%) of the health centres assessed had a functioning ambulance, its mismanagement was identified as a major deterrent to the referral system. The use of the ambulance for administrative purposes and the lack of budget for fuel delays referrals. Studies in developing countries also documented that, despite the availability of an ambulance, its mismanagement affects referrals [12,13]. The availability of an ambulance is not enough to ensure effective referral. Therefore, it needs effective management of ambulances, including availability of fuel, dedicated use of ambulances for emergencies, and drivers on standby 24/7.

The WHO obstetric and newborn care quality standard states that for every woman and newborn referred within or between health facilities, there should be appropriate information exchange and feedback to relevant health care staff [5]. However, effective feedback on cases referred has been generally low or ineffective in developing countries [14]. This study’s findings also showed that only 36% of the health centres received timely case-by-case feedback from hospitals (referral centres). Therefore, it is critical to improve referral feedback that could improve learning and accountability.

Health facilities must have a list of network referral facilities where there is a formal agreement for communication and feedback [5]. In this study, though 96% of the health centres had an up-to-date list of network referral health facilities, only 66% of the health centres had formal agreements and communication arrangements with referral centre(s). In addition, the findings show that a lack of referral guidelines creates misunderstandings with referral centers. Therefore, developing a referral guidline and having a formal memorandum of understanding with referral centres will improve referral feedback and understanding between the health facilities.

WHO estimates that about 15% of all pregnant women are expected to have life-threatening obstetric complications requiring referral. The 32% obstetric referral rate observed in this study substantially exceeded the WHO estimate [1,3]. Similarly, most studies in Ethiopia and other developing countries reported referral rates of 28–73%, which far exceeded the WHO estimate [7–10]. However, some studies also reported referral rates within the WHO estimate. A study in India reported the obstetric referral rate of 14% [15].

The qualitative findings of this study identified that the limited ability of the partograph to differentiate women with real-life-threatening obstetric complications contributes to the high referral rate. Similarly, WHO identified that the old partograph, which was in use in the study health centers during the study, had low specificity to diagnose obstetric complications. The WHO issued a new partograph that improves the ability of the partograph to identify women with true obstetric complications and reduce unnecessary referral and obstetric interventions [17,18]. Therefore, the health centers in Addis Ababa should adopt the new WHO labour guide and partograph for the management of labour.

Providers’ ability to timely and accurately diagnose obstetric complications and make urgent referral decisions plays a critical role in obstetric outcomes and reduces unnecessary referrals [1,5]. The qualitative finding in this study also attributed providers’ lack of skill to high referrals. A study in Eastern Ethiopia also documented that only 10% of all the obstetric referrals at referral centers were appropriate referrals, which shows the contribution of providers’ lack of skills to accurately diagnose obstetric complications to unnecessary referrals [11]. Therefore, reducing unnecessary referrals needs training providers on the obstetric and newborn care skills and adopting referral guidelines.

## Conclusion

Only a quarter of the health centers meet all eight standards for functional referral systems, and there is a high rate of obstetric referrals that far exceeds the WHO estimated referral rate. The referral system in the health centers is challenged with limited availability and management of ambulances, lack of formal agreements with referral centers, the absence of referral guidelines, and lack of timely feedback from referral centers, limited providers’ skills, and low specificity of the old partograph to identify women with true obstetric complications, and risk-averse practices driven by accountability pressures for unfavorable maternal and newborn outcomes.

Therefore, reducing unnecessary high referrals requires improving functionality of the referral system, especially ensuring emergency transport (ambulance) is available 24/7, and there is effective ambulance management, establishing formal agreements with referral centers and structured timely feedback mechanisms, adopting and training skilled birth attendants on the new WHO labour guide and new partograph, and instituting supportive accountability frameworks that is based on trust than fear of punishment.

## Supporting information

S1 File IRB clearance. (PDF)

S2 File Data. (SAV)

S3 File Human participants research checklist. (PDF)

S4 File Inclusivity questionnaire. (PDF)
