# Supplementary material for: Obstetric referral practices and health system factors in public health centres of Addis Ababa, Ethiopia: A mixed-methods study
Source: PLoS One. 2026 Aug 25;21(8):e0342872. doi: 10.1371/journal.pone.0342872 (PMC13505931; doi:10.1371/journal.pone.0342872)
Supplement: Supplementary file 1 [file pone.0342872.s001.pdf]

UNISA university of south africa

RESEARCH ETHICS COMMITTEE: DEPARTMENT OF HEALTH STUDIES
REC-012714-039 (NHERC)

5 February 2019

Dear Dr Abebe Haile Amaha

Decision: Approval

HSHDC/895/2019
Student: Dr Abebe Haile Amaha
Student No.:64041662
Supervisor: Prof RMM Mmusi Phetoe
Qualification: PhD
Joint Supervisor: -

Name: Dr Abebe Haile Amaha

Proposal: A strategy to improve quality of obstetric and new born care in Ethiopian Health Centres

Qualification: DPCHS04

Risk Level: Medium Risk

Thank you for the application for research ethics approval from the Research Ethics Committee: Department of Health Studies, for the above mentioned research. Final approval is granted from 5 February 2019 to 5 February 2024

The application was reviewed in compliance with the Unisa Policy on Research Ethics by the Research Ethics Committee: Department of Health Studies on. 5 February 2019

The proposed research may now commence with the proviso that:

1) *The researcher/s will ensure that the research project adheres to the values and principles expressed in the UNISA Policy on Research Ethics.*

2) *Any adverse circumstance arising in the undertaking of the research project that is relevant to the ethicality of the study, as well as changes in the methodology, should be communicated in writing to the Research Ethics Review Committee, Department of Health Studies. An amended application could be requested if there are substantial changes from the existing proposal, especially if those changes affect any of the study-related risks for the research participants.*

RECEIVED
2019 -02- 06
Executive Dean
College of Human Sciences

are substantial changes from the existing proposal, especially if those changes affect any of the study-related risks for the research participants.

3) *The researcher will ensure that the research project adheres to any applicable national legislation, professional codes of conduct, institutional guidelines and scientific standards relevant to the specific field of study.*

4) *You are required to submit an annual report by 30 January of each year that that he study is active. Reports should be submitted to the administrator HSREC@unisa.ac..az Should the reports not be forthcoming the ethical permission might be revoked until such time as the reports are presented.*

Note:

The reference numbers [top middle and right corner of this communiqué] should be clearly indicated on all forms of communication [e.g. Webmail, E-mail messages, letters] with the intended research participants, as well as with the Research Ethics Committee: Department of Health Studies.

Kind regards,

Prof JE Maritz
CHAIRPERSON
maritje@unisa.ac.za

Prof A Phillips
DEAN OF COLLEGE OF HUMAN SCIENCES

አዲስ አበባ ከተማ አስተዳደር ጤና ቢሮ
City Government of Addis Ababa Health Bureau

Ref.No ጀ/ጀስጦ/35934/227

Date 13/7/2017

- Gulelle Sub-city Health Office
- Kirkos Sub-city Health Office
- Nifas Silk Lafto Sub-city Health Office
- Arada Sub-city Health Office
- Yeka Sub-city Health Office
- Zewuditu Memorial Hospital
- Terunesh Beijing Hospital
- Bole Sub-city Health Office
- Kolfe Kernio Sub-city Health Office
- Lideta Sub-city Health Office
- Akaki Kality Sub-city Health Office
- Addis Ketema Sub-city Health Office

Addis Ababa

Subject: Request to access Health Facilities to conduct approved research

This letter is to support **Amaha Haile Abebe** to conduct research, which is entitled as "**A Strategy to Improve Quality of Obstetric and New-born Care in Ethiopian Health Centers..**" was duly reviewed and approved by Addis Ababa Health Bureau IRB, and the principal investigator is informed with a copy of this letter to report any changes in the study procedures and submit an activity progress report to the Ethical Committee as required. Therefore we request the Health facility and staffs to provide support to the Principal investigator.

With Regards

Kassayenew Amare

Ethical Clearance committee

Cc

- **Amaha Haile Abebe**
- **To Ethical Clearance Committee**

Addis Ababa
